# Time-variant left ventricle models for intracardiac impedance analysis

**DOI:** 10.2478/joeb-2024-0015

**Published:** 2024-10-05

**Authors:** Daniel Voss, Clara Wemmer, Steffen Leonhardt, Marian Walter

**Affiliations:** 1 Chair for Medical Information Technology, RWTH Aachen University, Aachen, Germany

**Keywords:** simulation, FEM, heart geometry, left ventricular volume, intracardiac impedance

## Abstract

Cardiovascular diseases are a leading cause of mortality worldwide. Thus, critically ill patients require continuous monitoring of cardiovascular indicators, such as the left ventricular volume (LVV). Although continuous hemodynamic monitoring of patients is desirable, due to technical limitations, current measurement technologies either require manual intervention of the physician or only provide inaccurate results. Intracardiac impedance measurements are a promising approach for continuous assessment of cardiac function. However, developing and evaluating these methods requires a simulation model of the left ventricle with cardiac motion during an entire cardiac cycle. While many models exist for a fixed ventricle size, to date, no freely available models incorporate time and represent the cardiac motion during a complete cardiac cycle. Therefore, we developed four cardiacmechanical left ventricular models with varying ventricle sizes and complexities. Each model focuses on a different aspect of the geometric shape, thus allowing an isolated analysis of the different influences. This paper presents the development of the models and initial results of the impedance analysis. All measured admittances exhibit a high resemblance for all models and a strong, non-linear correlation with the LVV. A comparison between the models shows how the different geometries affect the impedance. The models, thus, provide a useful basis for the development of LVV estimation algorithms.

## Introduction

Globally, cardiovascular diseases rank as the predominant cause of mortality across both developed and developing nations, with nearly 20 million individuals succumbing to these conditions in 2022 [1]. The precise and effective management of patients afflicted by these diseases mandates the continuous monitoring of critical parameters [2]. Among these, the left ventricular volume is recognized for its significant prognostic value in various cardiovascular pathologies, such as heart failure and myocardial infarction [3].

The pursuit of continuous monitoring is a dynamic field of research. Dating back to the 1950s, the technique of intracardiac impedance-based monitoring has been identified as a potential method for evaluating cardiac functionality [4]. This involves the placement of a multi-electrode catheter within the left ventricle (LV) to measure electrical impedance. Notably, electrical admittance exhibits a pronounced non-linear relationship with LVV. Study of the waveform morphology has not yet made clinical impact, however algorithms have been formulated to deduce LVV from admitance magnitude and phase based on simple models of the ventricle and analytical considerations.

Despite the introduction of numerous intricate algorithms since the 1980s [5, 6, 7, 8], their clinical application remains limited due to the extensive calibration they necessitate. The analytical algorithms developed by Baan and Wei, which are among the most frequently utilized, model the LV as an elongated cylinder, a simplification that overlooks the true geometry of the ventricle and thus demands manual calibration. The development of precise 4D heart models (3D+time) is critical for enhancing intracardiac impedance methodologies that require minimal or no calibration. Such models could facilitate the generation of algorithms derived from numerically simulated impedances, incorporating accurate heart geometry. However, the creation of these models is hindered by the scarcity of exact 4D representations of actual human hearts.

In response to this challenge, we have constructed four distinct heart models, each varying in complexity. The initial model adopts a cylindrical form, mirroring the designs of Baan [5] and Wei [6], to enable direct comparison. The second model expands upon the cylindrical concept to include the tapering at the apex. The third model accounts for the narrowing at both ends of the ventricle and the variations in ventricular length throughout the cardiac cycle. The final model is a physiological representation based on computed tomography (CT) data of a human heart, as documented by Unberath et al. [9]. All models are designed to simulate intracardiac impedance measurements using an electrode catheter, accommodating different LVVs. Initial impedance analyses for these models have also been performed.

This paper builds upon the preliminary results published in [10] and covers the design of four 4D ventricle models. Further, the simulation and impedance analysis are presented, illustrating the different advantages and drawbacks of each model.

## Methods

Cardiac-mechanical models have been developed to characterize cardiac motion, and finite element method (FEM) simulations of intracardiac electrical impedance have been performed. The left ventricle changes its shape throughout the cardiac cycle, influencing intracardiac impedance. This motion encompasses myocardial thickening and longitudinal and circumferential contraction, facilitating blood ejection into the aorta. These complex motions are simplified in the first three models in the modeling process to enable focused analysis of the distinct influences.

### Cylinder model

The cylinder model represents a simple approximation of the left ventricle, maintaining a uniform diameter throughout its height and thus disregarding the ventricle’s conical geometry. Nevertheless, the foundational equations for left ventricular volume estimation via impedance have been derived using this cylindrical assumption. Baan et al. formulated a conductance-to-volume conversion equation predicated on the cylindrical segments that collectively approximate the LV [5]. Furthermore, Wei et al. conducted an in-vitro assessment of the conductance catheter measurement system employing cylindrical vessels and numerical finite element models to emulate conductance catheter readings [11, 6]. Thus, the cylinder model is appropriate for the evaluation of these algorithms. The LVV of a cylinder of length l and radius *r* is given by

1 $${\text{LVV}}_{\text{cylinder}}=\pi r^{2}l.$$

The dominant factor contributing to volume changes of the ventricle is the alteration of its diameter. Prior research [12, 13] has shown that variations in ventricular length exert only a small influence. Hence, this change in length is neglected here and this model is kept at a fixed length of 90 mm. Thus, simulating the cardiac cycle with volumes ranging from 45 mL to 140 mL yields a diameter varying between 26 mm and 44mm.

In its basic form, as described in literature, this model lacks the aorta and myocardium. To enhance the model, a cylinder representing the aorta with a diameter of 30 mm is added and the ventricle itself is modeled by myocardium of constant thickness. Although myocardial thickness fluctuates with contraction and relaxation during the cardiac cycle, these variations are locationspecific, and comprehensive data reflecting myocardial thickness changes throughout the ventricle during the cardiac cycle are not available. Hence, the myocardium is represented with a consistent thickness of 12 mm.

**Figure 1:**
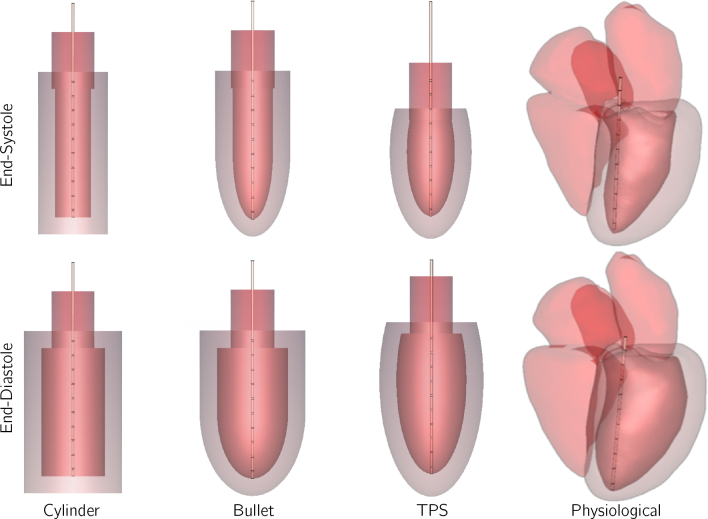
The four ventricle models. The blood is displayed in red and the ventricle is semitransparent red. The surrounding lung tissue is not shown.

The electrode catheter utilized for the simulated impedance measurements is positioned in the center of the model. A 7 F catheter (outer diameter of 2 mm) equipped with 10 electrodes, each 1 mm wide and spaced at 10 mm intervals from each other, is incorporated into the model. To prevent current injection into the myocardium, the bottom electrode is situated 5 mm from the apex, while the top electrode is placed just above the aortic valve. The first column of Figure 1 depicts the final model in both the end-systolic and end-diastolic phases.

### Bullet model

The bullet model offers a more accurate representation of the LV’s morphology. This model is frequently utilized for LVV calculations in 2D-echocardiography methods [14, 15, 16]. It integrates a cylindrical upper section with an ellipsoidal lower segment, mirroring the tapering apex of the LV, with each segment accounting for half of the LV’s total length. Consequently, the ellipsoidal segment has two minor axes with the same length as the radius *r* and a major axis measuring half the total length *l*. The LVV for the bullet model can be expressed by

2 $${\text{LVV}}_{\text{bullet}}=\frac{5}{6}\pi r^{2}l.$$

The simulation parameters mirror those of the cylinder model. The LV is modeled with a constant length of 90 mm, while the diameter, derived from eq. (2), varies between 28 mm and 48 mm over the cardiac cycle. The myocardium enveloping the model has a thickness of 12 mm. Additionally, the model incorporates a cylindrical aorta, 30 mm in diameter. The catheter is positioned partially within the aorta, aligned with the longitudinal axis. Given the model’s fixed length, the uppermost electrode is consistently situated above the aortic valve for the duration of the cardiac cycle. The resultant model, captured at both end-systole and end-diastole, is illustrated in the second column of Figure 1.

### Truncated prolate spherical model

The truncated prolate spherical model (TPS) introduces a variation in ventricle length. Initially proposed by Domingues et al. [17] as a mathematical model for determining the surface area of the human LV, this model has been applied to compute LVV from cardiac imagery. It was subsequently validated in bovine subjects for predicting LVV for volumes reaching 164 mL [18].

The application of the TPS model for intracardiac impedance simulations, as in this study, is not documented in existing literature. Schiller et al. [19] have delineated methods for gauging LV dimensions using two-dimensional echocardiography. They suggest dividing the TPS model’s long axis into segments measuring 2/3 *l* and 1/3 *l*. With a short axis length of *r*, the TPS model’s LVV is given by

3 $${\text{LVV}}_{\text{TPS}}=3\pi r^{3}.$$

The resulting model at end-systole and end-diastole phases is shown in the third column of Figure 1. In this model, the LV length varies between 67 mm and 98 mm, and the diameter ranges from 34 mm and 50 mm throughout the cardiac cycle. The length, thus, exceeds the typical physiological range of 84 mm to 93 mm [13]. Despite this, the TPS model is advantageous in evaluating the impact of catheter positioning shifts along the longitudinal axis. The myocardium encasing the model is again portrayed with a uniform thickness of 12 mm. The catheter is anchored at the apex, akin to patient measurements, which results in electrode displacement relative to the aortic valve as volumes vary, potentially extending into the aorta. The aorta is once more represented as a cylinder with a 30 mm diameter.

### Physiological model

The physiological model is based on image data of a human heart. It has been created from images at 5 distinct time points and thus is only available at discrete time steps of the cardiac cycle. Besides the changes in volume, the model incorporates changes of ventricle length, inner diameter, and myocardium thickness. Thus, this model is the most realistic, but also more complex. In the following the model creation process and the resulting model parameters are presented.

The physiological model has been created from CT images of a heart. Data were taken from the opensource 4D statistical shape model of the whole heart published by Unberath et al. [9]. This model is one of few open-source models that includes data at different time points of the cardiac cycle and was originally developed as a numerical phantom for cone-beam CT simulation. It contains surface meshes from ten-phase angiography measurements of the aorta, atria, ventricles, and left ventricular myocardium at ten phases during the cardiac cycle derived from data of 20 patients. The model is implemented in the author’s open-source simulation and reconstruction framework CONRAD. A metric volume phantom was created using the multi-material phantom renderer and exported in TIF format. The image processing package Fiji, a distribution of ImageJ, was used to generate the surface meshes. Segmentation of the blood inside the four chambers, left ventricular myocardium, and the aorta was done by thresholding. This approach extracts objects from the background by classifying pixels into a group whose intensities are larger than a specific value. To avoid holes in the final model, individual thresholds were used for each compartment. Autodesk Meshmixer© and the software Open Flipper were used to remove holes in the structures and for surface smoothing. The final surface meshes of the models were imported into the FEM simulation tool CST Studio as STL files and inserted with the following procedure to remove overlapping portions. First, the blood of the LV, left atrium, and aorta was added, so that these structures form one solid. Afterwards, the created left heart blood was inserted into the myocardium. The resulting structure was inserted into the right heart, which consists of the right atrium and ventricle. The resulting model is depicted in the fourth column of Figure 1.

The model embodies both left and right ventricles, which are filled with blood, and the ascending aorta all depicted in red. The LV is surrounded by the semitranslucent red left ventricular myocardium. The catheter is placed with the tip at the apex and the top extending into the aorta into the LV. This results in an off-center position close to the ventricular wall.

Throughout the cardiac cycle, the LVV varies between 73 mL and 146 mL, resulting in a stroke volume of 73 mL. Myocardial thickness ranges between 8 mm and 16 mm. The left ventricular inner diameter fluctuates between 40 mm and 50 mm, while the length varies from 81mm to 90 mm.

### Volume cycle

For the simulation of the LV geometry during the cardiac cycle a reference volume is needed. To ensure the results’ comparability across all models, a uniform volume cycle was adopted. Since the physiological model is only available at fixed steps, the volume cycle of this model is used as the reference volume.

**Figure 2:**
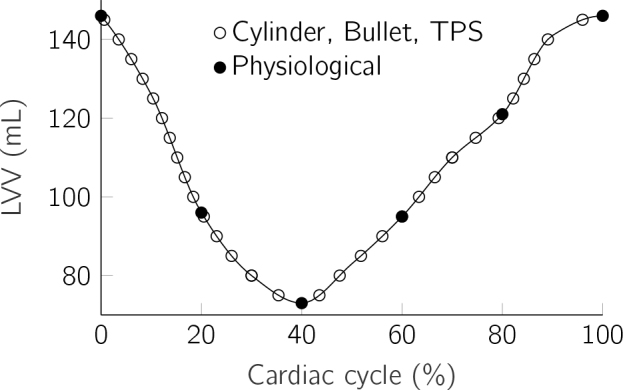
LVV during one cardiac cycle. The first three models are simulated for volume increments of 5 mL, while the physiological model is only available for the 5 indicated volumes.

The image data of the physiological model are available for ten discrete time points during the cardiac cycle. However, due to the extensive manual labor involved in modeling and simulation, only half of these time points are utilized for the simulation of the physiological model. For the volume reference, the LVV at all 10 time steps has been evaluated and a shape-preserving piecewise cubic interpolation was applied. The resulting volume cycle is shown in Figure 2, with the values at each of the ten available phases marked by a circle. The analytical models are continuously available and can be simulated at any volume of the reference curve.

### Electrical properties

The simulation of the electrical fields requires the electrical properties of each model component. The simulation models comprise of four materials: blood, myocardial tissue, the surrounding tissue, and the catheter. Both, the left ventricle and the aorta contain blood, the ventricle is modelled by myocardium. The surrounding matter is modeled by lung tissue, reflecting the heart’s primary anatomical enclosure. Each material is presumed to be homogenous and anisotropic.

The simulated impedance measurements were conducted at 50 kHz. The electrical properties of blood, myocardium and background tissue at this frequency are derived from Gabriel et al. [20]. The electrical conductivities *σ* for blood, myocardium, and lung tissue are then respectively 0.7 S/m, 0.19 S/m, and 0.1 S/m. The associated relative permittivities *ϵ_r_* are given by 5197, 16982, and 4272, correspondingly.

Additionally, the catheter is modelled with perfectly conducting electrodes connected by polyimide sections with conductivity *σ* = 2 × 10^-8^ S/m and permittivity *ϵ_r_ =* 3.52.

### Ethical approval

The conducted research is not related to either human or animal use.

## Results

To simulate an impedance measurement, simulations with an injection of electrical current in the outermost electrodes have been performed for each model. The physiological model has been simulated at the available volume points, while the other three models have been simulated in increments of 5 mL. The electrical field has been determined from the FEM simulation in CST Studio using the low frequency solver. Subsequently, the transfer impedance and admittance for various electrode configurations were deduced from the field distribution.

**Figure 3:**
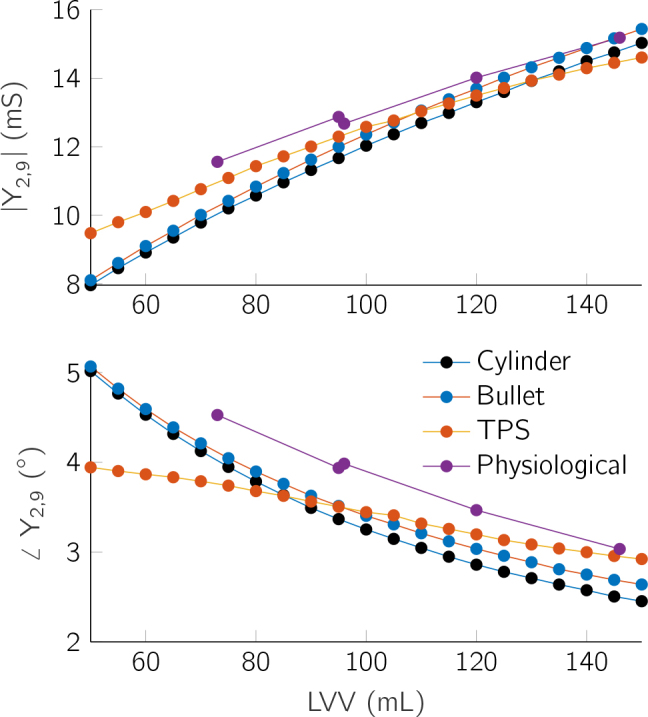
Simulated admittances show a non-linear correlation with the LVV. Admittance magnitude (top) and admittance phase (bottom).

**Figure 4:**
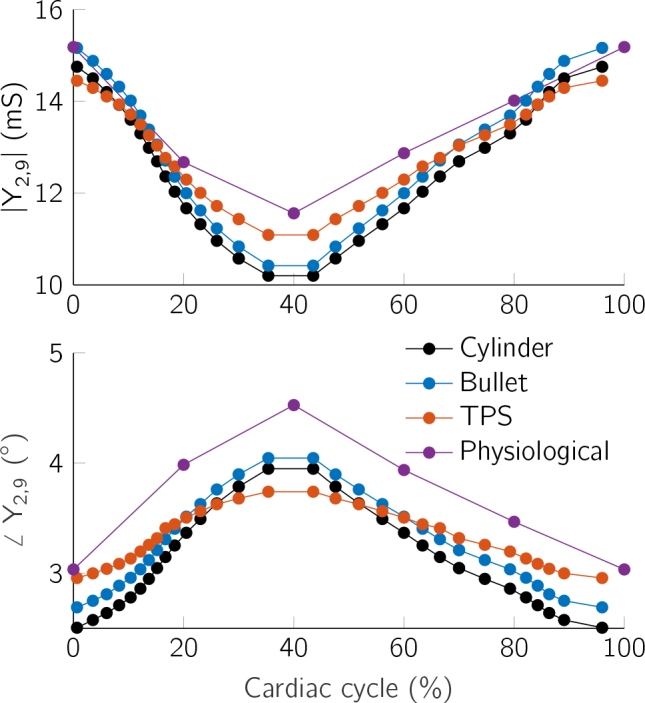
Simulated admittances correlate strongly with the LVV throughout a cardiac cycle. Admittance magnitude (top) and admittance phase (bottom).

The transfer admittance *Y*_2,9_ is measured with a 4 electrode measurement using the outermost electrodes for current injection and the neighboring ones for measurement. Figure 3 shows the admittance measurement over the LVV. Figure 4 shows the admittance measurement throughout a cardiac cycle.

A substantial correlation between transfer admittance and LVV was observed across all models. The correlation is non-linear and positive for admittance magnitude and negative for the phase. The cylinder and bullet models demonstrated similar admittance profiles, which aligns with expectations given their structural resemblance. In contrast, the TPS and physiological models showed less variation in admittance throughout the cardiac cycle. The TPS model’s admittance alterations, both in magnitude and phase, were minimal due to the shifting relative electrode positions in relation to the ventricle as the volume changed, thus only being partially influenced by the volume alteration. The small variation in the admittance magnitude of the physiological model is due to the off-center position of the catheter, which further results in a stronger impact of the higher permittivity of the myocardium, thus displaying the highest phase angle of all models for a given LVV. This maximum phase angle of 4.6 ° is only exceeded by the other models for very small LVVs when the myocardium is very close to the catheter.

## Discussion

The developed 4D cardiac mechanical ventricle models allow for an extensive analysis of the geometric influences on cardiac impedance. The ability to simulate the LV’s motion throughout a cardiac cycle offers a dynamic approach to understanding heart function and impedance changes.

The four different models allow for a comprehensive analysis of various geometric influences on cardiac impedance. Overall, the transfer admittance showed a high similarity between the models, but differed due to the individual shapes of the models and the catheter placement. The cylinder and bullet models differ only in the shape of the apex. The simulated transfer admittances show the strongest similarity, thus highlighting the difficulty in reliably estimating the volume in the apex. Consequently, LVV estimation algorithms based on a cylinder model, such as by Baan [5] and Wei [6], cannot reliably estimate the LV without correction factors.

The human heart changes its length during the cardiac cycle which has a significant impact on the measured admittance, as shown for the TPS model. Therefore, a change in LV length should be included in models used to obtain intracardiac admittance data. The change in length of the TPS model is greater than in a real heart. Nevertheless, the strong influence shows that a volume estimation algorithm has to account for the longitudinal catheter position and needs reliable positioning at the aortic valve.

The physiological model is influenced by all of these factors and is additionally impacted by the off-center catheter placement, close to the ventricle wall. Nevertheless, this complex model shows an overall similar trend to the simplified models. While the simplifications in the models allow for isolated analysis, they may not capture the full complexity of the heart’s geometry, potentially affecting the accuracy of the results. A systematic variation of the electrical properties and catheter offset to more accurately determine the impact of each influence is the topic of further research. Nevertheless, the obtained simulation results correlate with real measurements described in literature [21, 6]. However, additional validation against clinical data is necessary to confirm their reliability and practical application in real-world scenarios, while also using a more accurate LVV cycle that corresponds closely to the clinical data.

The major barrier in obtaining a clinical dataset for comparison is the need for a high quality reference, because all available measurements, such as MRI or echocardiography, have also limitations in accuracy. Further, since the position of the catheter is essential the catheter movement has to be recorded as well.

## Conclusion

The 4D cardiac mechanical ventricle models represent a promising tool for advancing cardiac care and offer a useful foundation for developing LVV estimation algorithms. The design of the first three models mimics the heart’s morphology, yet deliberate simplifications allow for targeted analysis of disparate factors influencing impedance. In contrast, the more anatomical physiological model allows for a more realistic assessment of cardiac impedances, although it offers only limited time and volume steps. The similarity between the cylinder and bullet models indicates that accurately predicting the volume in the apex based on intracardiac impedance measurements is challenging. This suggests that an algorithm should include prior information regarding the geometry. Moreover, these models permit a focused examination of different influencing factors, including the catheter offset and different electrode configurations, as well as a comparison of diverse conductance and admittance algorithms, which is the subject of future work. Therefore, these models pave the way for the developement of more sophisticated cardiac monitoring technologies, which have the potential to improve patient outcomes.
